# Randomized controlled single-farm field evaluation of an autogenous *Mycoplasma hyosynoviae* bacterin: lameness treatments and exploratory serology

**DOI:** 10.1186/s40813-026-00503-7

**Published:** 2026-03-20

**Authors:** Marit Gaastra Maaland, Marianne Oropeza-Moe

**Affiliations:** Department of Production Animal Clinical Sciences, Norwegian University of Life Sciences, Sandnes, Norway

**Keywords:** Fattening pigs, Lameness, Swine health, Arthritis, *Mycoplasma hyosynoviae*, Autogenous vaccine

## Abstract

**Background:**

*Mycoplasma hyosynoviae* arthritis contributes to antimicrobial use and reduced welfare in fattening pig production. No commercial vaccines are available, and the efficacy of autogenous vaccines remains poorly documented. This study aimed to evaluate the effect of an autogenous *M. hyosynoviae* vaccine on lameness occurrence, describe *M. hyosynoviae* antibody profiles over time, and assess such profiles in relation to lameness development.

**Case presentation:**

**Methods**. A randomized, blinded, controlled field trial was conducted in a Norwegian fattening pig herd and its associated piglet-producing herd. Piglets received two intramuscular injections of either vaccine or saline at six and nine weeks of age. Lameness occurrence was monitored throughout the fattening period, and serum samples from a subset of pigs (n=118) were collected at 6, 8, 13, and 19–21 weeks of age for detection of *M. hyosynoviae*-specific IgG antibodies by T20 IgG ELISA. Data were analyzed using logistic regression models.

**Results:**

A total of 737 pigs (383 vaccinated, 354 controls) were evaluated for lameness. The incidence of lameness treatments was 7.8% (95% CI: 5.1–10.5) in the vaccine group and 6.8% (95% CI: 4.2–9.4) in controls, with no significant difference between groups (OR=1.28, 95% CI: 0.76–2.17, p=0.354). Most lameness cases occurred during the first three weeks after arrival at the fattening farm. Vaccination was not associated with increased odds of serum IgG seropositivity by the T20 ELISA in an exploratory subset (OR=0.97, CI: 0.36–2.58, p=0.947). Antibody levels varied with sampling time; the highest levels were observed at the end of fattening.

**Conclusions:**

Autogenous vaccination with a *Mycoplasma hyosynovia*e bacterin at six and nine weeks of age was not associated with a measurable reduction in lameness treatments in this study, and vaccinated and control pigs showed similar T20 IgG ELISA antibody profiles. Results should be interpreted in light of study limitations, including diagnostic uncertainty, factors related to vaccination timing and early immune dynamics, and the exploratory nature of the serological analysis. These findings highlight the challenges of evaluating autogenous *M. hyosynoviae* vaccines in the field and underscore the need for further research on effective preventive strategies.

**Supplementary Information:**

The online version contains supplementary material available at 10.1186/s40813-026-00503-7.

## Background

*Mycoplasma hyosynoviae*, a commensal of the upper respiratory tract thought to be widespread globally, can cause arthritis in pigs older than ten weeks of age [1–3]. Reported morbidity in infected herds ranges from less than 1% to 50% [2, 4]. While acute arthritis usually resolves within three to ten days, chronic cases may also occur [2, 4]. Clinically affected pigs may exhibit stiffness, shifting lameness, non-weight-bearing of a limb or an inability to rise, most often involving the hind legs [5, 6]. The pain associated with *M. hyosynoviae-*arthritis constitutes a significant welfare issue in affected animals [5]. Reduced mobility and lack of appetite can lead to decreased feed intake, weight loss and impaired growth performance [3, 6]. In addition, the labor and cost related to clinical surveillance, antibiotic and NSAIDs treatments and culling of lame animals can have negative economic impacts for pig producers [4, 5].

Antimicrobial therapy is commonly administered for the treatment of acute *M. hyosynoviae*–associated arthritis in growing pigs, thereby contributing to overall antimicrobial use in affected herds [5, 7, 8]. Reducing stress has been suggested as a general preventive measure, but the implementation of specific control strategies is hampered by limited understanding of disease pathogenesis [3, 6, 9]. Vaccination could offer an alternative preventive approach, potentially reducing antimicrobial use and improving animal welfare; however no commercial vaccines against *M. hyosynoviae* are currently available. A vaccine developed in Denmark showed promising results under experimental conditions but failed to prevent lameness in a field trial [10].

Autogenous vaccines tailored for use in individual herds are employed in some countries and are anecdotally reported to be effective, although published evidence supporting their efficacy is generally lacking [3, 7]. Two recent conference abstracts described an effect of *M. hyosynoviae* autogenous vaccination; the first reported a reduction in culling rate of gilts in a German farm, while the second observed a lower risk of developing lameness in vaccinated pigs in a large U.S. herd [11, 12].

The primary objective of the present study was to evaluate the efficacy of an autogenous *M. hyosynoviae* vaccine in preventing lameness in a Norwegian fattening pig herd. Secondary objectives were to describe and compare *M. hyosynoviae* IgG antibody development over time in vaccinated and control animals, and to assess these antibody profiles in relation to lameness development in the herd.

## Case presentation

### Materials and methods

#### Farms, housing and management

The field study was conducted between November 2023 and June 2024 at a fattening farm and its associated piglet-producing farm, located approximately 13 km apart in Rogaland County, Southwestern Norway. The Norwegian pig population is free from several major porcine pathogens, including porcine reproductive and respiratory syndrome virus, pseudorabies virus, transmissible gastroenteritis virus, porcine epidemic diarrhea virus, swine influenza viruses (except H1N1[pdm09]) and *Mycoplasma hyopneumoniae* [13]. Apart from lameness in the fattening herd, no specific disease problems were reported during the study period.

The farms were selected based on anticipated farmer compliance and previous clinical and treatment history. In the fattening herd, 20–30 pigs per batch of approximately 280 animals were routinely treated with tiamulin due to *M. hyosynoviae*-associated lameness. Prior diagnostic investigations of several pigs from two batches had confirmed *M. hyosynoviae*-associated arthritis through necropsy and histopathological evaluation of joint tissues, routine bacteriological culture of synovial fluid, and multiplex PCR testing for *M. hyosynoviae*, *Mycoplasma hyorhinis*, *Streptococcus suis*, and *Glaesserella parasuis* on synovial membrane or fluid samples. All diagnostic procedures were performed at the Norwegian University of Life Sciences (NMBU), except multiplex-PCR analyses, which were conducted at a commercial laboratory abroad.

The piglet-producing farm operated in a 5.5-week batch farrowing system with 45 sows per batch, producing approximately 5500 piglets annually. The sows were TN70 (Topigs Norsvin 70 x Yorkshire Z-line) hybrids inseminated with Duroc semen. Breeding animals were routinely vaccinated with a combination vaccine against porcine parvovirus and *Erysipelothrix rhusiopathiae* (Porcilis^®^ Ery Parvo vet, MSD Animal Health Norge AS), and against *Escherichia coli* (Porcilis^®^ Porcoli Diluvac Forte vet, MSD Animal Health Norge AS). Suckling piglets were vaccinated against porcine circovirus type 2 (Ingelvac^®^ CircoFLEX, Boehringer Ingelheim Animal Health Nordics AS). The herd practiced free farrowing in individual farrowing pens comprising a solid floor area with sawdust bedding material, and a slatted floor area. Cross-fostering was performed during the first days of life. In compliance with Norwegian legislation, male piglets were surgically castrated by a veterinarian using local anaesthesia and long-acting systemic analgesia (NSAID), and no tail docking was performed [14]. After weaning at approximately 34 days of age, piglets were moved to a nursery room where two litters were mixed in each pen, with about 18 piglets per pen. The smallest piglets from several litters were housed together in separate pens. Each pen consisted of a solid floor covered with sawdust bedding and a slatted floor area. Floor heating in the pens was set to 22 °C at start and gradually reduced to 18.5 °C. Piglets were sold in groups to three different fattening farms once they reached approximately 33 kg, at different time points depending on piglet weight. The first group, comprising 280 piglets, was sold at around 10 weeks of age to the fattening farm included in this study.

The fattening farm produced approximately 1800 pigs for slaughter per year and was managed by the farmer and one employee. The farm consisted of a single building divided into two units, each housing one age group. Each unit contained two animal rooms with twelve pens (12.5 m^2^ each), accommodating up to twelve pigs per pen. Piglets were separated by sex upon arrival. Room temperature was maintained at 20 °C upon entry and gradually decreased to 14 °C. Pens included a solid concrete floor area partial floor heating and sawdust bedding, and a slatted floor area. Twice daily, pens were inspected, cleaned, and fresh sawdust bedding material added to the solid floor area. Straw and/or shredded paper was provided as rooting material. Sick pens were available in each unit for pigs requiring additional care. Pigs were fed a commercial dry diet ad libitum and had unrestricted access to adequate quantities of water. The facility was mechanically ventilated using fans and a perforated ceiling. Each batch was sent to slaughter at 20–24 weeks of age in sub-groups based on weight. Pens were cleaned with cold water and left empty for 1–2 weeks between batches.

#### Vaccine production

In March 2023, three fattening pigs (weighing 37, 42, and 50 kg) exhibiting hind limb lameness were euthanized on-farm and transported to NMBU for necropsy. Synovial fluid was aseptically collected from a total of 19 joints using a sterile needle and syringe following skin removal and flame sterilization of the joint capsule area. Samples were immediately transferred into mycoplasma transport medium (Copan Universal Transport Medium^®^, Copan Italia S.p.a., Brescia, Italy) and shipped to a commercial laboratory (Austrian Agency for Health and Food Safety Ltd. (AGES) Animal Health, Mödling, Austria) for culture. Gross and histological lesions from all three pigs were consistent with *M. hyosynoviae*-associated arthritis.

A total of 16 *M. hyosynoviae* isolates were successfully cultivated from the three pigs. As previously described [15], multi-locus sequence typing (MLST) of three isolates (one per pig) revealed that all isolates belonged to sequence type 58 and formed a distinct clonal cluster when compared to isolates from Austria, Germany, Denmark and the USA. The isolates were subsequently shipped to a commercial facility (AniCon, SanGroup Biotech Germany GmbH, Höltinghausen, Germany) for vaccine production. Three isolates (one per pig) were selected for inclusion in the vaccine and propagated to 3.2–5.0 × 10^8^ CFU/ml, yielding a final bacterial concentration of 3.45 × 10^8^ CFU per 2-ml dose prior to inactivation. Formaldehyde was used for inactivation, and an oil-in-water formulation (O/W), consisting of mineral and non-mineral oil, was used as adjuvant to enhance vaccine immunogenicity. The finished vaccines were transported to NMBU in temperature-controlled packaging and stored at 4 °C, protected from light, until use. All vaccines were administered within their designated shelf life.

#### Experimental design

A randomized, blinded, controlled trial was designed to assess the effectiveness of a two-dose autogenous *M. hyosynoviae* vaccine.

The farmer reported treating 20–30 pigs per batch for presumed *M. hyosynoviae*-associated lameness, corresponding to 7–11% morbidity. Sample size calculation was based on 80% power, a two-sided significance level of 0.05, equal group sizes, and an expected lameness incidence of 7% in the control group and 2% in the vaccine group. A low level of lameness was anticipated in the vaccine group due to other causes that may be clinically indistinguishable from *M. hyosynoviae*-associated arthritis (e.g., osteochondrosis, or arthritis of other bacterial origin). The calculation indicated a required total of 610 animals (305 per treatment group). For practical reasons, and to allow for potential losses to follow-up (as only the first 280 piglets from each batch were sent to the study fattening farm), approximately 300 piglets per batch were included from three consecutive batches.

At the piglet-producing farm, piglets were ear tagged shortly after birth with unique identification (ID) numbers starting from 1 and counting upwards. Treatment allocation (vaccine or control) was based on the last digit of the individual ID number: per pen, all piglets with odd numbers received one treatment, and those with even numbers received the other. A computer-generated randomization list determined which sequence (odd=vaccine or even=vaccine) applied to each pen. Both treatment groups were represented in every pen. A small number of piglets pre-selected by the farmer as replacement gilts were neither vaccinated nor saline-injected. To ensure inclusion of treated animals in the batch transferred to the fattening farm, pens containing the largest piglets were pre-selected for the study.

Prior to trial initiation, vaccine safety was assessed in ten non-study piglets, which were injected with the vaccine and observed for one hour by researchers and daily by the farmer for five days; no adverse reactions were noted. During the study, all piglets were observed for immediate post-vaccination reactions and general health. Piglets received either 2 ml of vaccine (vaccine group) or 2 ml of 0.9% sterile saline (control group) intramuscularly in the neck area at six and nine weeks of age. The vaccination schedule was determined based on the vaccine manufacturer’s experience and the reported typical onset of lameness in the fattening herd (one to three weeks post-arrival), as no scientifically validated guidelines were available.

Piglets were transported to the fattening farm at approximately ten weeks of age. Each batch of piglets was transported to the fattening farm on a single truck and unloaded simultaneously, with pigs exiting in a continuous sequence. Each batch filled two rooms, with pens stocked sequentially from the unloading flow across both rooms (se details in Additional file 1). Within rooms, females were placed in pens on one side of the central corridor and males on the other side. Pigs were not pre-sorted by treatment group prior to placement. The outcome “presumed *Mycoplasma hyosynoviae*–associated lameness” was assessed in the fattening herd and defined as lameness affecting one or more legs (excluding foot or claw lesions) that warranted antimicrobial treatment with tiamulin (15–25 mg/kg/day). Prior to study initiation, farm staff received detailed instructions on lameness assessment and recording procedures. Both farm staff and investigators were blinded to treatment allocation throughout the study.

Lameness surveillance was conducted twice daily (morning and afternoon). All pigs were observed rising and moving within the pen. Pigs showing stiffness or subtle lameness after initial movement were moved to the hallway for further observation of gait. The case definition used by farm staff was a pig that either required assistance to rise, was non-weightbearing or clearly relieving a limb, or displaying pronounced stiffness with stride shortening, or visible lameness after exercise. No ordinal scoring scale was used. Animals with lesions of the foot or claw were excluded from the case definition. Pigs meeting the case definition criteria were treated with tiamulin. Severely lame pigs or pigs that developed other illnesses were moved to sick pens and not mixed back. Lame pigs were followed up-daily and treatment was repeated if lameness persisted on the day(s) following initial treatment, based on clinical reassessment. Severely lame animals that did not recover within three to five days were euthanized. A researcher participated in lameness evaluation together with farm staff on two occasions, during the first and second batches of pigs. Inter-observer variability was not formally assessed. Lameness events were recorded using a standardized scoring sheet that included date, pig identification, room and pen number, affected leg(s), antimicrobial used, dose, and duration of treatment. All pigs receiving antimicrobial treatment or euthanized on farm were recorded, regardless of clinical signs.

#### Serology

A subset of 20 vaccinated and 20 control pigs per batch (120 pigs in total; convenience sample of 1–2 pairs of vaccinated and control animals per pen) was selected for blood sampling. No formal sample size calculation was performed for the serological analyses. The primary purpose of this component was to describe antibody kinetics over time following vaccination under field conditions, rather than to detect small between-group differences in antibody levels. Accordingly, serology analyses should be considered exploratory. Samples were collected on the same day but prior to the first vaccination at six weeks of age, and subsequently from the same animals at 8, 13, and 19–21 weeks of age. Serum samples were stored at -20 °C until shipment to a commercial laboratory (Veterinary Diagnostic Lab, Iowa State University, Ames, USA) that recently developed a T20 IgG ELISA for detection of *M. hyosynoviae*-specific antibodies. The original validation study reports diagnostic sensitivity and specificity across a range of cut-off values but does not provide intra- or inter-assay coefficients of variation [16]. Seropositivity was determined using a sample-to-positive (S/P) cutoff value of 0.5, according to the criteria established by Giménez-Lirola et al. [16]. Each serum sample was analysed once; assays were not performed in duplicate.

#### Data analysis

Clinical and laboratory data were compiled in Microsoft Excel (Microsoft Corporation, Redmond, WA, USA) and analyzed using Stata (Stata SE18, StataCorp LLC, College Station, Texas, USA). Lameness incidence was calculated for the vaccine and control groups as the number of pigs treated for lameness divided by the total number of pigs per group. 95% confidence intervals (CI) were computed for each proportion, while inferential comparisons were performed using logistic regression with room included as a fixed effect and pen-level clustering. Only antimicrobial treatments administered for pigs meeting the case definition were included in the lameness endpoint. The primary endpoint was first lameness treatment only, and repeat events were not included in analysis. Pigs removed from the study before the end of the fattening period for reasons unrelated to lameness were considered random drop-outs. These animals were excluded from the final analysis after best-case and worst-case sensitivity analyses confirmed that their exclusion did not affect the study conclusions. To account for the possibility that cases of lameness occurred before protective immunity from the booster vaccination could be established, a sensitivity analysis was performed in which pigs treated for lameness within (i) 0–14 days, and (ii) 0–21 days after booster vaccination were excluded.

Antibody responses (positive/negative) were analyzed using mixed-effects logistic regression because serostatus was assessed repeatedly within the same pigs across four sampling times, leading to within-animal correlation and violation of the independence assumption of standard logistic regression. Vaccination group and sampling time were included as fixed effects, and their interaction was assessed to evaluate whether vaccine effects differed over time. A random intercept for pig was included to account for clustering of repeated observations within pigs and to model between-pig heterogeneity in baseline odds of seropositivity. Model results are presented as odds ratios (OR) with 95% CI, and statistical significance was set at *p* < 0.05.

### Results

**Fig. 1:**
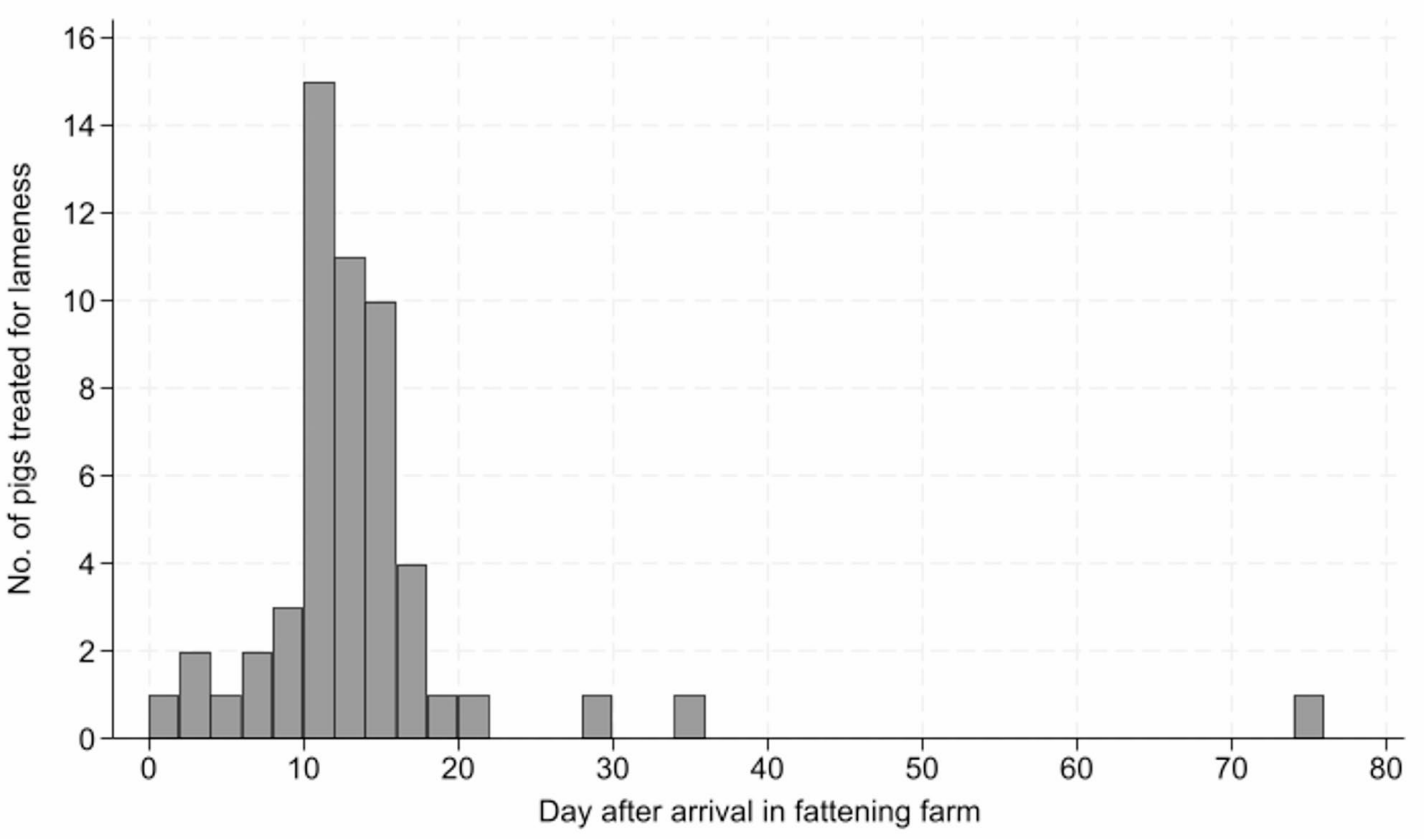
Lameness occurrence in the fattening farm. Days from arrival in the farm to first *M. hyosynoviae*-associated lameness treatment is shown in 54 pigs across experimental treatment groups

A total of 737 pigs were evaluated for lameness throughout the fattening period, including 383 vaccinated and 354 control pigs. Lameness incidence was 7.8% (95% CI: 5.1–10.5) among vaccinated pigs and 6.8% (95% CI: 4.2–9.4) among control pigs. Vaccination was not associated with a reduced odds of lameness (OR = 1.28, 95% CI: 0.76–2.17; *p* = 0.354). No significant effect of room was detected. Accordingly, no statistically significant difference in lameness incidence was observed between vaccinated and control pigs. Five pigs in the control group and one in the vaccine group were euthanized on-farm for reasons unrelated to lameness and were excluded from the analysis. Four additional pigs lost their ear-tags upon lameness occurrence and were also excluded. Most lameness cases occurred within the first three weeks after arrival at the fattening farm (Fig. 1). The proportion of lameness cases occurring 0–14 days, 15–21 days, or more than 21 days after booster vaccination was 7.4%, 29.6% and 63.0%, respectively. Sensitivity analyses excluding cases occurring (i) 0–14 days or (ii) 0–21 days after the booster dose did not materially change the estimated treatment effect (OR = 1.32, 95% CI: 0.77–2.26, *p* = 0.314 and OR = 1.32, 95% CI: 0.80–3.07, *p* = 0.191, respectively).

Of the 58 pigs treated for lameness, 46 recovered after a single dose of tiamulin. Seven pigs required a second dose on the following day, and one pig received treatment for three consecutive days. One animal was euthanized due to chronic lameness.

Regarding limb involvement, the right and left hind legs were affected in 46.3% and 40.7% of cases, respectively. Lameness involving both hind legs, the right front leg, or all legs occurred in 5.6%, 5.6%, and 1.9% of cases, respectively. Following apparently successful treatment of right or left hind limb lameness, four pigs experienced recurrence 15–22 days later, involving the same limb (*n* = 2) or both hind legs (*n* = 2).

**Fig. 2:**
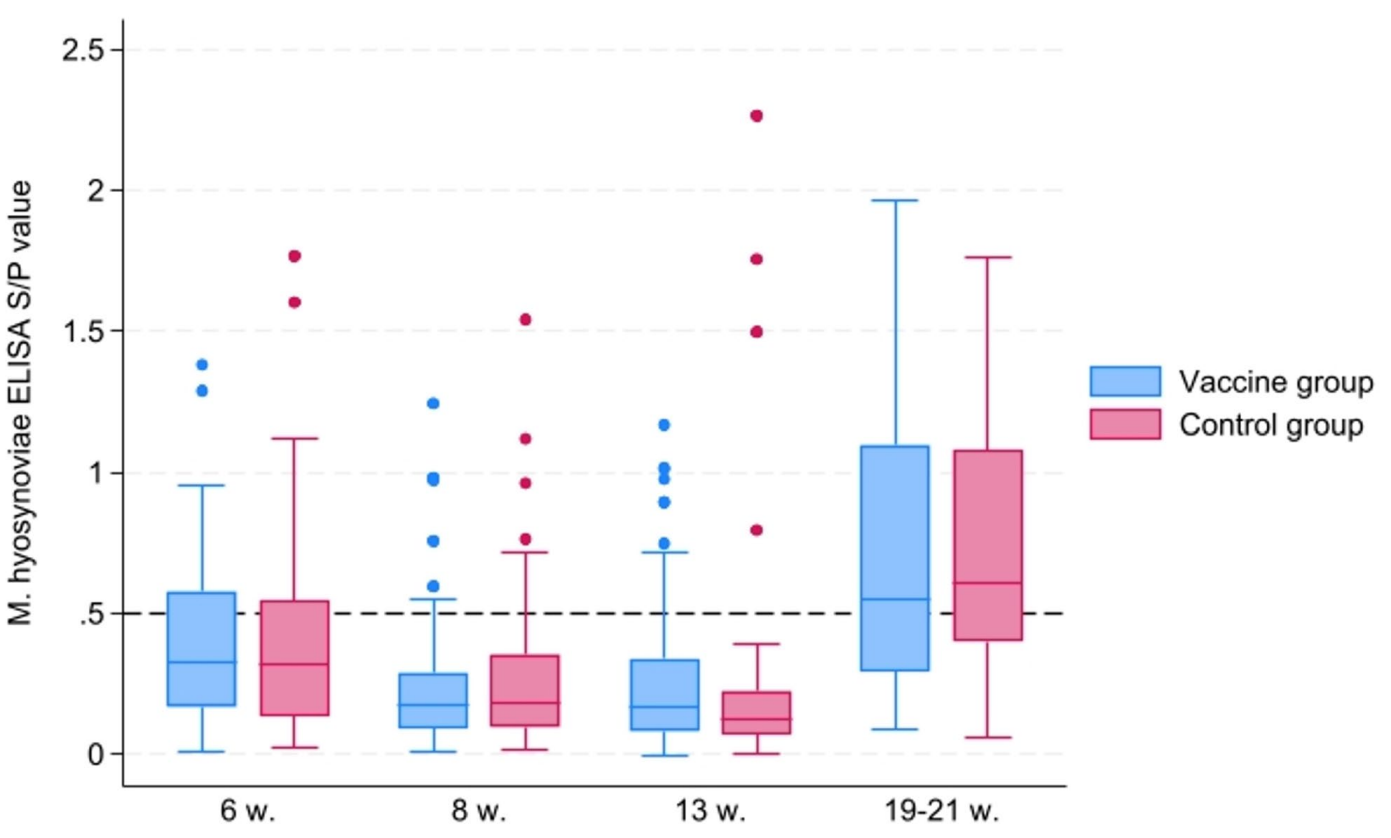
*M. hyosynoviae* T20 IgG ELISA antibody development over time in vaccinated and control pigs. S/P values are depicted for vaccinated (blue) and control (red) pigs at 6, 8, 13, and 19–21 weeks of age (w). The dashed horizontal line denotes cut-off for seropositivity (S/P value ≥ 0.5)

For the evaluation of *M. hyosynoviae* antibody responses, 118 pigs were included, contributing a total of 430 serum samples collected across four sampling points (Table 1; Fig. 2). Mixed-effects logistic regression analysis showed no significant effect of vaccination group on the likelihood of IgG antibody positivity across all time points (OR = 0.97; 95% CI: 0.36–2.58; *p* = 0.947). In contrast, sampling time had a significant effect on antibody response. Compared with six weeks of age, the odds of antibody positivity were significantly lower at eight and 13 weeks of age (OR = 0.33, 95% CI: 0.12–0.92, *p* = 0.035 and OR = 0.14, 95% CI: 0.04–0.52, *p* = 0.003, respectively), but markedly higher at 19–21 weeks of age (OR = 3.95, 95% CI: 1.50–10.43, *p* = 0.006). Interaction terms between vaccination group and time were not statistically significant (all *p* > 0.2), indicating that the effect of vaccination did not differ between sampling times. The estimated variance of the random effect for pig was 1.40 (95% CI: 0.58–3.38). The corresponding ICC (latent-variable method) was approximately 0.30, suggesting moderate within-pig correlation of serostatus across repeated samples. Pigs lost to follow-up included those that died or were euthanized, lost their ear-tags, or were not transferred to the fattening pig farm.

**Table 1:** No. and percentage of *M. hyosynoviae* antibody positive pigs per age and treatment group

	6 w.		8 w.		13 w.		19–21 w.	
	Vac	Con	Vac	Con	Vac	Con	Vac	Con
No. positive (% positive)	18 (30.0)	18 (31.0)	6 (10.0)	9 (15.8)	9 (16.1)	4 (8.9)	27 (51.9)	25 (59.5)
Total no.	60	58	60	57	56	45	52	42

Cut-off for *M. hyosynoviae* IgG ELISA seropositivity: S/P value ≥0.5. Vac: vaccine group, Con: control group, w: weeks of age

**Fig. 3:**
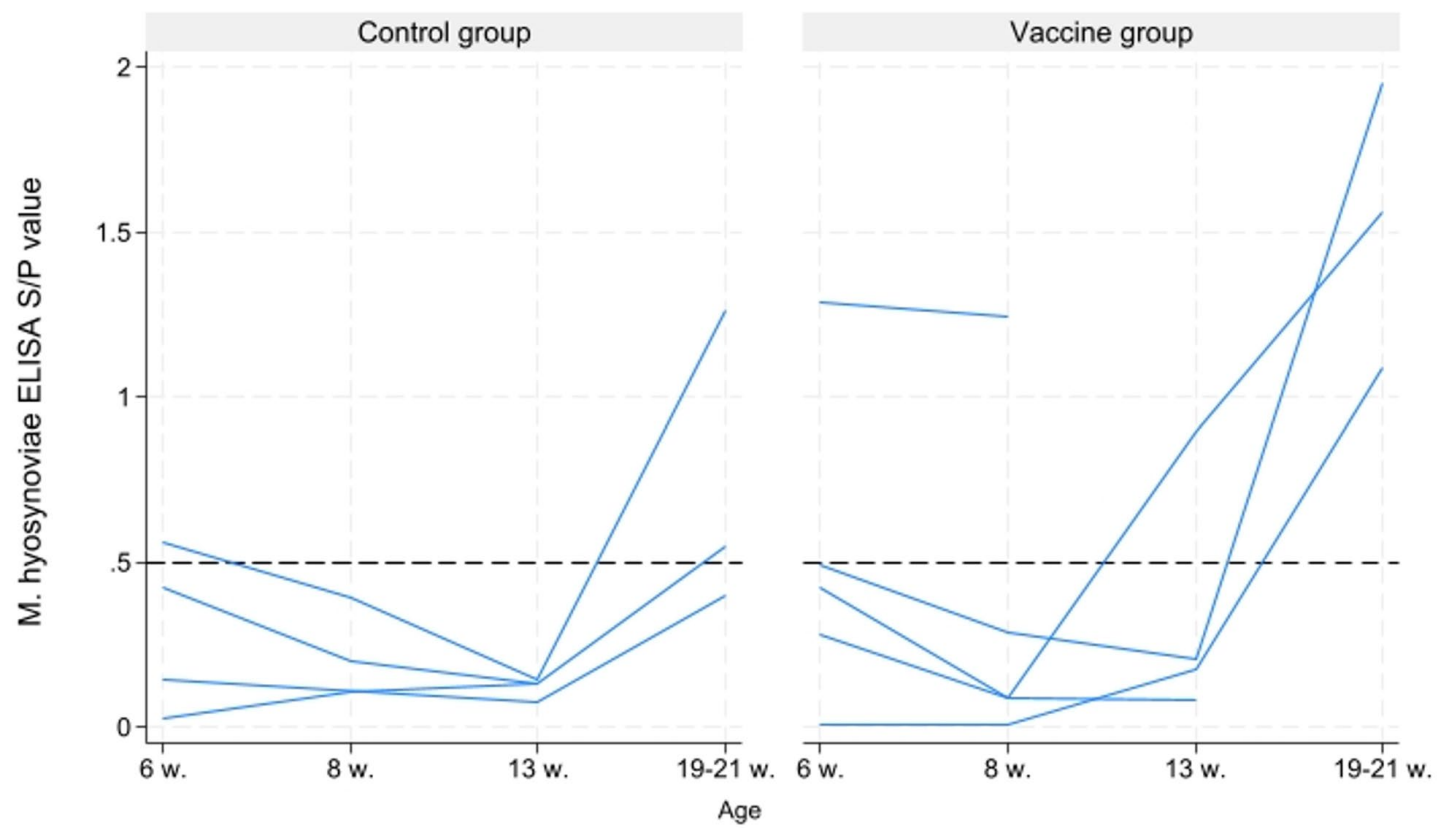
*M. hyosynoviae* T20 IgG ELISA antibody development in lame pigs. S/P value development over time is depicted in nine pigs treated for lameness at 10–12 weeks of age and belonging to either vaccine or control group. The dashed horizontal line denotes cut-off for seropositivity (S/P value ≥ 0.5)

Five vaccinated pigs and four controls included in the blood sampling subset were treated for lameness at 3 (*n* = 1), 11 (*n* = 6), 12 (*n* = 1) or 14 (*n* = 1) days after arrival at the fattening farm (corresponding to 10–12 weeks of age). With few exceptions, these pigs were *M. hyosynoviae* IgG negative until 13 weeks of age, after which they developed detectable T20 IgG ELISA antibody levels (Fig. 3). One pig did not develop a measurable antibody response throughout the study. Three pigs were lost to follow-up after the second or third sampling point.

## Discussion and conclusions

Autogenous vaccination against *M. hyosynoviae* did not prevent the occurrence of lameness in this herd, which contrasts with previous reports demonstrating a reduced lameness-related culling rate of gilts [11] or reduced risk of lameness development in finishers [12]. However, the present findings are consistent with those of a Danish field trial evaluating a novel *M. hyosynoviae* vaccine, in which vaccination or placebo injection of piglets two and five weeks post-weaning did not affect lameness development [10]. Direct comparison with these studies is limited by the restricted information available in published conference abstracts.

The lack of vaccine efficacy observed in this study may be attributed to technical or biological factors of the vaccine. Technical issues could include manufacturing errors or inadequate adjuvant performance [17, 18], which were not further investigated. It should also be noted that the quality of autogenous vaccine products may differ between manufacturers and countries [19]. Biologically, incompatibility between the bacterial strains used in the vaccine and the field strains circulating during the trial may have contributed [18]. To account for the potential presence of multiple pathogenic strains, vaccine isolated were obtained from three lame pigs; all isolates, however, belonged to the same sequence type and clonal cluster [15], making this explanation less probable. Nevertheless, a shift in the predominant field strain between bacterial isolation and trial initiation eight months later cannot be excluded, as *M. hyosynoviae* isolation and characterization from lameness cases during the trial were not performed.

Other factors that may have contributed to the lack of vaccine efficacy include the timing of vaccination relative to the onset of lameness, potential interference with maternal antibodies, and early colonization with *M. hyosynoviae* [20]. The second vaccine dose was administered at nine weeks of age, while lameness was observed in some pigs as early as ten weeks of age, leaving a narrow interval for the development of protective immunity. Following bacterin vaccination, seroconversion and maturation of an effective immune response typically require more than one week, with antigen-specific IgG often increasing over several weeks following prime-boost autogenous vaccination in swine [21, 22], suggesting that the vaccination schedule may not have allowed sufficient time for immunity to develop before clinical disease occurred. Sensitivity analyses excluding cases occurring either within 0–14 days or within 0–21 days after the booster dose did not materially change the estimated treatment effect. These findings suggest that early post-vaccination cases were unlikely to have masked a clinically relevant vaccine effect, but the possibility that the vaccination schedule did not allow sufficient time for development of protective immunity cannot be excluded.

Vaccinated and control pigs were housed together within pens at the finisher farm, meaning that indirect effects of vaccination on transmission dynamics cannot be excluded. If vaccination reduced bacterial shedding or transmission from vaccinated pigs, exposure pressure for control pigs may also have been reduced, potentially diluting any measurable vaccine effect. This potential “spillover” effect should therefore be considered when interpreting the absence of a detected treatment effect.

*M. hyosynoviae* maternal antibodies have been reported to persist for 8–12 weeks, and it has been proposed that colostrum-derived antibodies and potentially other immune-factors, including cellular components such as primed lymphocytes, at least partially protects piglets against lameness before ten weeks of age [23, 24]. Whether these maternal immune factors also may interfere with vaccine-induced immunity remains unknown. In the present study, approximately 30% of pigs possessed *M. hyosynoviae*-specific T20 IgG ELISA antibodies at six weeks of age followed by a decline in levels at eight and 13 weeks of age, suggesting passive transfer through colostrum. Early colonization cannot be excluded, however, as pigs may have been exposed to or colonized by *M. hyosynoviae* prior to vaccination. Although transmission of *M. hyosynoviae* in young piglets appear to be fairly rare, colonization may occur before six weeks of age [23, 25]. Tonsillar colonization has been associated with a serological response [26], and recent work has shown that vaccination does not prevent tonsillar colonization [27]. The serological data therefore may reflect maternally derived antibodies, early exposure, or a combination of these, which may have limited the potential impact of vaccination on subsequent clinical disease. As the relative contributions of maternal antibody and early natural exposure could not be distinguished in this study, any inference regarding potential interference with vaccine responsiveness should be considered hypothesis-generating.

The antibody profile of vaccinated pigs in this study did not differ from that of control animals, indicating that vaccination failed to elicit an antibody response detectable by the T20 IgG ELISA. The serology subset was, however, not designed to detect small between-group differences in antibody responses between vaccinated and control pigs, and the convenience sampling approach is one of several key limitation of the study; other limitations include single-run testing without duplicates, and lack of reported intra- and inter-assay coefficients of variation. While the data allow descriptive assessment of antibody dynamics over time, definitive conclusions regarding vaccine immunogenicity cannot be drawn from this component alone. Interpretation of the serological findings should also consider the characteristics and intended use of the T20 IgG ELISA; this research-use assay has demonstrated a high diagnostic specificity (100%) and sensitivity (96.8%) at a S/P value cutoff of ≥ 0.5 [16], but no gold standard exists for defining protective antibody responses against *M. hyosynoviae*. In this context, the relative contributions of humoral and cell-mediated immunity to protection against *M. hyosynoviae* remain poorly characterized [3]. Available evidence suggests that cell-mediated immune mechanisms may play a role in protection, whereas the protective significance of circulating antibodies is less clear and may be variable [23, 28]. Consequently, the absence of antibodies detectable by the T20 IgG ELISA alone should not be interpreted as evidence of immunological failure.

Antibody development patterns in *M. hysoynoviae*-infected herds with or without lameness have previously been described in both Denmark and the U.S. In one Danish study, eight herds exhibited heterogeneous antibody profiles with no consistent relationship to clinical status [26]. Another Danish investigation reported a clear serological response in fattening pigs from a herd affected by *M. hyosynoviae*-associated arthritis, whereas no such response was observed in two unaffected herds [23]. Conversely, two non-clinical U.S. herds showed increasing antibody levels throughout the fattening period, while antibody levels remained low in a clinical herd [29]. Although differences in sampling times and serological assays complicate direct comparison, the antibody pattern observed in our study aligns most closely with those reported for the non-clinical U.S. herds and both clinical and non-clinical Danish herds. Overall, as no consistent between-herd pattern in antibody profiles has been demonstrated, the relationship between herd-level antibody data and clinical lameness remains unclear.

Antibody development in individual lame pigs following natural infection has not been previously documented. In this study, most pigs remained T20 IgG ELISA antibody-negative until the onset of lameness at 10–12 weeks of age, after which they developed a detectable antibody response. However, antibodies were also detected in one pig at eight weeks of age, while another showed no serological response throughout the fattening period. These findings should be interpreted with caution as the number of animals included was limited and some were lost to follow-up.

A principal limitation of this study was the lack of diagnostic confirmation of *Mycoplasma hyosynoviae* in lame pigs during the study period. Because no joint samples were collected and no PCR or culture was performed during the trial, the proportion of lameness cases truly attributable to *M. hyosynoviae* is unknown. Therefore, the results should be interpreted as applying to lameness cases requiring treatment rather than to confirmed *M. hyosynoviae* arthritis. The presumption of *M. hyosynoviae* involvement was based on prior herd diagnostic findings. Specifically, 10 pigs with clinical lameness were previously examined by necropsy during 2021–2023, of which eight pigs had lesions consistent with mild to severe non-purulent synovitis. Five of the cases were confirmed as positive for *M. hyosynoviae* and negative for other agents by multiplex PCR (*M. hyosynoviae*, *M. hyorhinis*, *S. suis*, and *G. parasuis*) and routine bacteriology, while three cases were negative by both methods. One pig had fibrinopurulent arthritis with a co-infection of *Actinobacillus pleuropneumoniae* and *M. hyosynoviae*, while no etiological agent was detected from one pig with purulent arthritis. In the present study, lameness cases were therefore classified as presumed *M. hyosynoviae*–associated based on clinical presentation alone, recognizing that lameness is not specific to this pathogen and may result from other infectious or non-infectious causes. In addition, lameness identification relied on standardized clinical observation by farm staff, which may have contributed to outcome misclassification. The etiologic uncertainty thereby represents a key limitation for causal interpretation of vaccine efficacy in this study. Future studies incorporating pathogen-specific diagnostics and objective outcome measures would improve outcome specificity and strengthen conclusions regarding vaccine efficacy against confirmed *M. hyosynoviae* disease.

In conclusion, vaccination of piglets with an autogenous *M. hyosynoviae* bacterin at six and nine weeks of age was not associated with a measurable reduction in lameness during the fattening period in this herd. Vaccinated and control pigs showed similar antibody profiles, but interpretation of the serological findings is limited by the exploratory nature of the serology subset and the absence of established correlates of protection. The lack of a detectable vaccine effect should be interpreted in light of study limitations, including the absence of pathogen-specific diagnostic confirmation, potential outcome misclassification, and uncertainties related to vaccination timing, early colonization, and maternal immunity. Overall, these findings highlight the challenges of evaluating autogenous *M. hyosynoviae* vaccines under field conditions and underscore the need for further controlled studies with improved diagnostic and immunological resolution, as well as complementary studies employing alternative study designs to better capture vaccine effects.

## Supplementary Information

Below is the link to the electronic supplementary material.

Supplementary Material 1

## Data Availability

The datasets used and/or analysed during the current study are available from the corresponding author on reasonable request.

## Abbreviations

°C: Degrees Celcius
CFU/ml: Colony-forming units per milliliter
CI: Confidence interval
ELISA: Enzyme-linked immunosorbent assay
H1N1[pdm09]: Hemagglutinin1 neuramidase1 pandemic 2009
IgG: Immunoglobulin G
kg: Kilograms
m^2^: Square meters
*M. hyosynoviae*: *Mycoplasma hyosynoviae*
OR: Odds ratio
PCR: Polymerase chain reaction
TN70: Topigs Norsvin 70 x Yorkshire Z-line
U.S.: United States
